# A Sub-millimeter, Inductively Powered Neural Stimulator

**DOI:** 10.3389/fnins.2017.00659

**Published:** 2017-11-27

**Authors:** Daniel K. Freeman, Jonathan M. O'Brien, Parshant Kumar, Brian Daniels, Reed A. Irion, Louis Shraytah, Brett K. Ingersoll, Andrew P. Magyar, Andrew Czarnecki, Jesse Wheeler, Jonathan R. Coppeta, Michael P. Abban, Ronald Gatzke, Shelley I. Fried, Seung Woo Lee, Amy E. Duwel, Jonathan J. Bernstein, Alik S. Widge, Ana Hernandez-Reynoso, Aswini Kanneganti, Mario I. Romero-Ortega, Stuart F. Cogan

**Affiliations:** ^1^Draper, Cambridge, MA, United States; ^2^Department of Neurosurgery, Massachusetts General Hospital, Harvard Medical School, Boston, MA, United States; ^3^Department of Psychiatry, Massachusetts General Hospital, Harvard Medical School, Charlestown, MA, United States; ^4^Picower Institute of Learning and Memory, Massachusetts Institute of Technology, Cambridge, MA, United States; ^5^Department of Bioengineering, University of Texas, Richardson, TX, United States

**Keywords:** wireless neural stimulation, implantable neurostimulators, electroceuticals, inductive coupling, microcoil

## Abstract

Wireless neural stimulators are being developed to address problems associated with traditional lead-based implants. However, designing wireless stimulators on the sub-millimeter scale (<1 mm^3^) is challenging. As device size shrinks, it becomes difficult to deliver sufficient wireless power to operate the device. Here, we present a sub-millimeter, inductively powered neural stimulator consisting only of a coil to receive power, a capacitor to tune the resonant frequency of the receiver, and a diode to rectify the radio-frequency signal to produce neural excitation. By replacing any complex receiver circuitry with a simple rectifier, we have reduced the required voltage levels that are needed to operate the device from 0.5 to 1 V (e.g., for CMOS) to ~0.25–0.5 V. This reduced voltage allows the use of smaller receive antennas for power, resulting in a device volume of 0.3–0.5 mm^3^. The device was encapsulated in epoxy, and successfully passed accelerated lifetime tests in 80°C saline for 2 weeks. We demonstrate a basic proof-of-concept using stimulation with tens of microamps of current delivered to the sciatic nerve in rat to produce a motor response.

## Introduction

Wireless neural stimulators are being developed to avoid complications associated with traditional lead-based implants (Sahin and Pikov, 2011). These complications include lead-breakage, scar-tissue growth, MRI restrictions, and undesirable tethering during animal studies (Hamani and Temel, 2012; Desai et al., 2015; Ersen et al., 2015). The smallest wireless stimulators developed to date are passive in nature and are powered electromagnetically from outside the body. This includes radio-frequency powered devices, such as non-radiative inductive coupling (Loeb et al., 2001) or mid-field energy transfer (Ho et al., 2014), as well as optically powered devices, such as near-infrared radiation (Abdo et al., 2011). The radio-frequency powered neural stimulators tend to be considerably larger than the optically-powered devices. However, the challenge with optically-powered devices is that light penetrates very poorly through tissue, allowing only superficial nerve targets. As a result, there is a need for radio-frequency powered stimulators that are on the submillimeter scale (<1 mm^3^) to allow for deeper nerve targets. Stimulators on this size-scale would allow the electronics and antenna to be entirely integrated into a nerve cuff for peripheral nerve stimulation, and could enable wireless deep brain stimulation.

The size of a wireless neural stimulator is often limited by wireless energy transfer, which necessitates antennas that are several millimeters in diameter to operate the device. Passive digital CMOS receivers that have been developed for inductively powered medical implants require at least 1 V to be induced in the implanted coil, which requires coils of at least 1 mm in diameter (Cho et al., 2013; Lee and Ghovanloo, 2013). Low-threshold FETs may allow the required voltage levels to be reduced, but at the expense of reliability (e.g., dropped bits). In order to minimize the amount of voltage that is required to operate an inductively powered neural stimulator, we have developed a simple design that consists only of an antenna to receive inductive power, a diode for rectification, and two electrodes on each of the device for current to flow through in order to excite neurons. Previous studies have explored similar concepts of direct rectification of the received signal, but the devices tend to be large (>1 cm on the longest dimension) (Ha et al., 2012; Towe et al., 2012).

Another factor that influences the size of the implant, aside from the antenna, is the packaging. Implants are commonly made using ceramic or titanium containers that are hermetically-sealed to protect the electronics from the body, and vice-versa. These containers tend to be too large, for example, to integrate onto a nerve cuff. Therefore, we chose to pursue a polymer-based encapsulation in order to maintain a compact form-factor.

**Figure 1:**
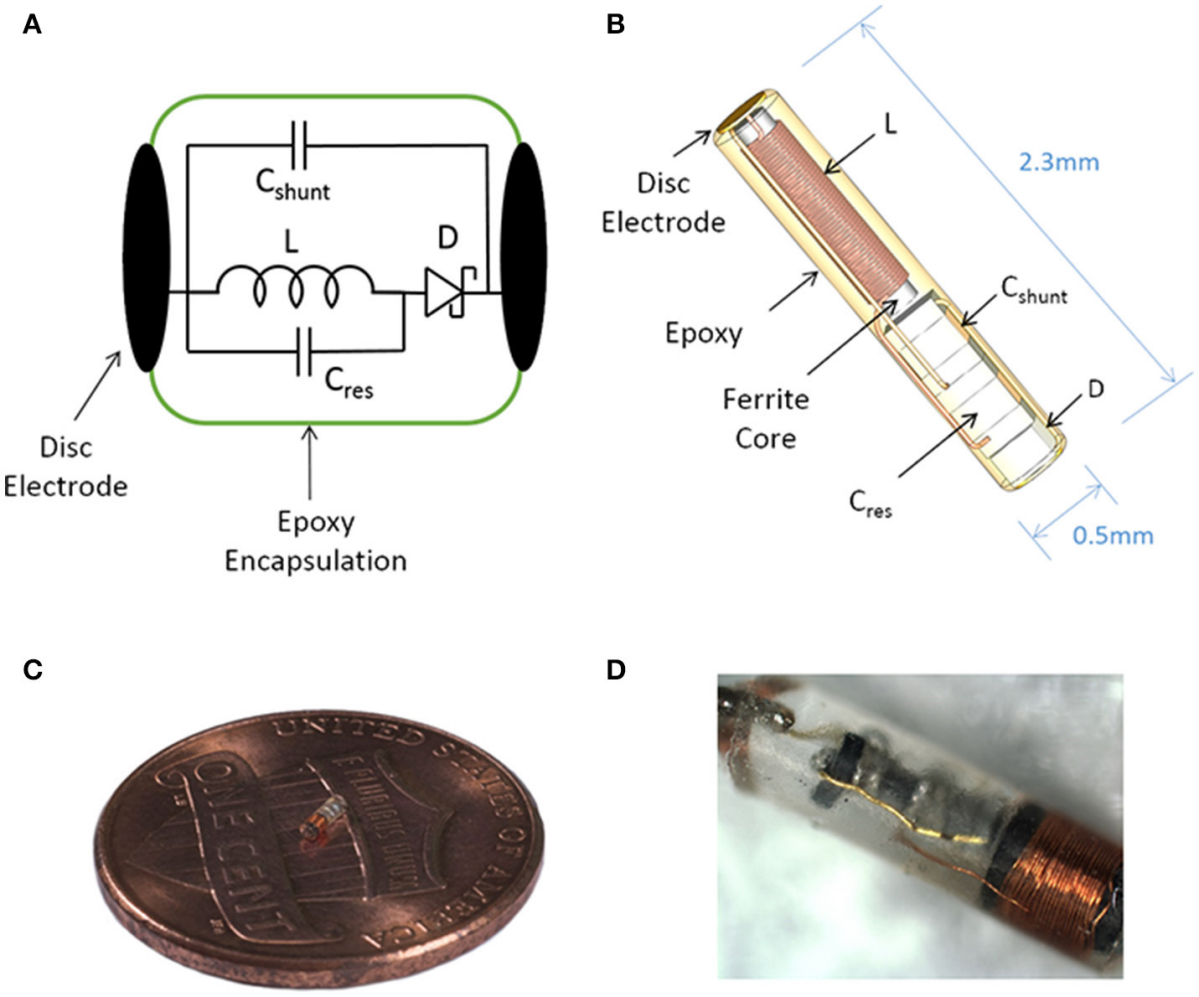
Illustration of the sub-millimeter, wireless stimulator. **(A)** The device consists of a coil (L) to receive inductive power, a capacitor (C_res_) for resonating the inductor, a Schottky diode (D) for rectification, and a shunt capacitor (C_shunt_) to facilitate rectification. **(B)** Assembly of the stimulator, with a total volume of 0.45 mm^3^. **(C,D)** Images of a built prototype.

Our goal was to design and build an inductively powered wireless neural stimulator that is sub-millimeter scale (<1 mm^3^) and can deliver sufficient current for excitation of a peripheral nerve. We present an analytical and computational model to help define the limits on antenna size. We report on a working prototype of the wireless neural stimulator (Figure 1). While this device offers stimulation only at a single site, as compared to multi-point stimulation devices, we believe the small size can offer a worthwhile tradeoff for certain applications in neural stimulation therapies.

## Methods

In order to define how much voltage and power is required to operate the device, it is first necessary to define (1) the limits of electromagnetic field exposure, (2) the electrical load of the tissue, and (3) how much current is needed to produce neural excitation.

### Defining limits for exposure to electromagnetic fields

In the United States, the safe level of radio-frequency (RF) exposure is defined by the FCC (see IEEE Std. C95.1; IEEE International Committee on Electromagnetic Safety, 2005). The standard metric used to define safe levels of RF exposure is the specific absorption rate (SAR). SAR measures the amount of RF energy that is absorbed in the body and converted to heat and is expressed in W/kg. The SAR limit for an occupational environment, such as a hospital, is defined as 8 W/kg, while the limits for an uncontrolled environment is 1.6 W/kg (Psathas et al., 2014) averaged over 1 g of tissue.

**Figure 2:**
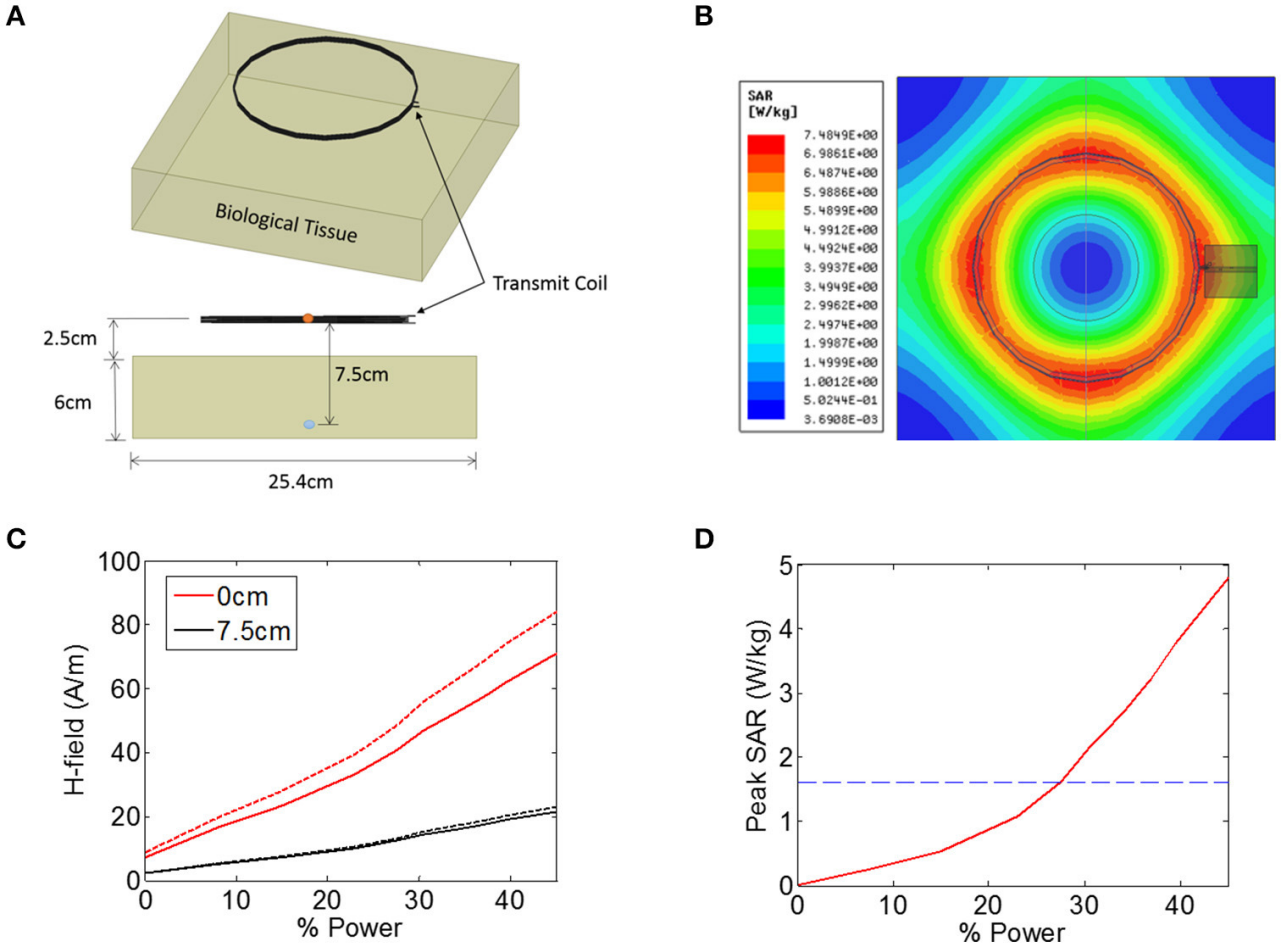
**(A,B)** Illustration of the finite-element model in ANSYS that was used to estimate the heating of biological tissue in response to an electromagnetic signal from a 15.2 cm diameter coil positioned 2.5 cm from the tissue. The magnetic field intensity was measured in the center the transmit coil (orange circle) and also 7.5 cm from the transmit coil (blue circle). **(C)** Magnetic field (H-field) vs. input power at the center of the transmit coils (0 cm, red solid line), as well as 7.5 cm from the transmitter (black solid line). The measurement was also made when the tissue was not present (dashed lines). **(D)** Peak SAR level simulated across the entire tissue as a function of input power, indicating the maximum allowed SAR level of 1.6 W/kg (blue dashed line).

To estimate SAR as a function of the applied time-varying electromagnetic field, full-wave simulations were conducted in ANSYS Electronics Desktop 2016. We used a model that consists of a four-loop transmit coil of 15.2 cm diameter that is positioned 2.5 cm above biological tissue measuring 25.4 × 25.4 × 6 cm (Figures 2A,B). Increasing the size of the tissue volume did not impact the results. Current was driven into the coil at a frequency of 10 MHz using a capacitive T-matching network to transform the inductance of the coil into a 50 Ω input impedance. A frequency of 10 MHz was chosen because the ferrite core in our device becomes lossy at frequencies of >10 MHz. In terms of the electrical properties of the tissue, we used conductivity and relative permittivity values of 0.5 S/m and 100, respectively, representing the approximate value of muscle, fat, and skin as measured at 10 MHz (Gabriel et al., 1996; Foster, 2000). Permittivity was taken to be a real-valued number rather than complex in order to represent the case where there is no dielectric loss due to polarization of the tissue.

The transmit power was varied across a range of power levels, as measured in the percentage of total power available from a benchtop power amplifier (Model AG 1021, T&C Power Conversion). The magnetic field intensity was noted at two specific locations: at the center of the transmit coil, and 7.5 cm axially into the tissue (5 cm beneath the surface of the tissue) (Figure 2C). The measurement was also taken when no tissue was present (Figure 2C, dashed lines), indicating that the presence of the tissue resulted in a minor attenuation of the applied magnetic field. In the animal experiments discussed later, the animal is placed on a non-conductive plastic table. Our simulations showed that this table had negligible impact on the magnetic field intensities present at the wireless stimulator (not shown).

In addition to measuring the magnetic field intensity, we also measured the SAR level averaged over 1 g of tissue (Figure 2D), showing that the limit for uncontrolled environments of 1.6 W/kg is reached for a power level of 27%, while the limit for the controlled environment of 8 W/kg was not encountered for the field levels tested here. It is important to note that the location within the model tissue at which the maximum heating occurred is not necessarily the same location as the implanted device. The goal of these simulations was to determine the magnitude of the magnetic and electric fields at the location of the stimulator while the maximum heating itself can occur in any location in the tissue.

### Defining the electrical load

In order to determine the level of current that is delivered by the device for a given amount of induced voltage in the implanted coil, it is necessary to define the electrical load of the tissue. When current is delivered to neural tissue with an electrode, there are two general sources of impedance: the electrode-electrolyte interface, and the impedance of the tissue itself. The tissue impedance contains both resistive and capacitive elements, but for bulk tissue, the resistive component is much lower impedance than the capacitive component (Reilly, 1992), and therefore the tissue is usually considered as a purely resistive medium (Gabriel et al., 1996, 2009; Foster, 2000).

**Figure 3:**
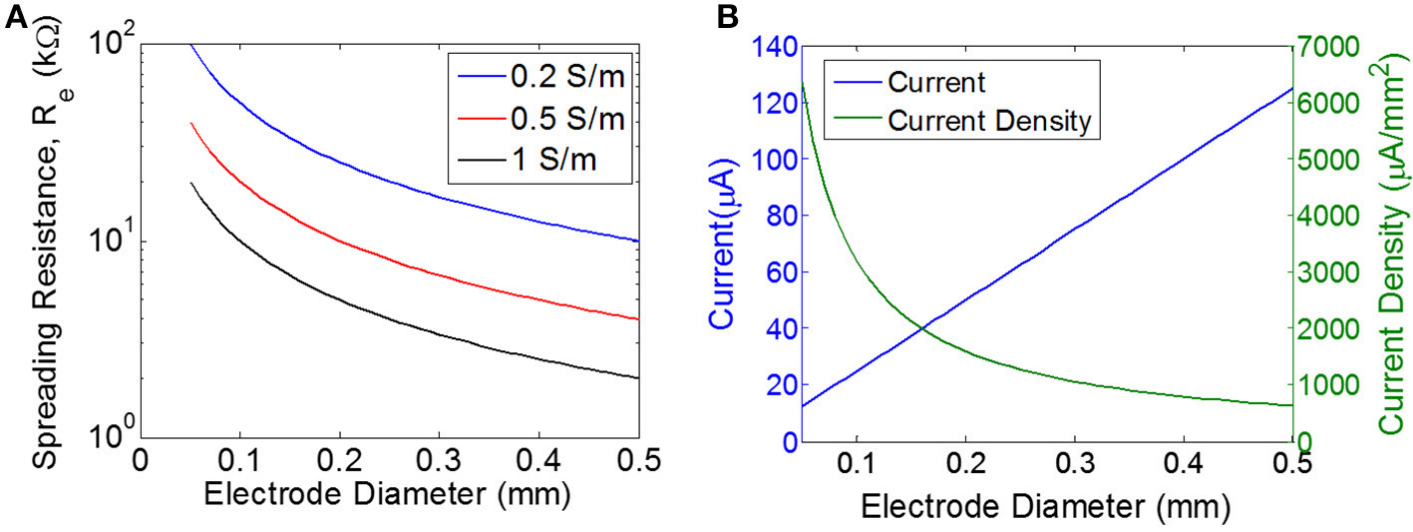
Estimating the load and the required current. **(A)** Spreading resistance of the tissue for a single electrode (R_e_) of varying diameter for three different tissue conductivities. **(B)** Current and current density for a single disc electrode of varying diameter, assuming input voltage of 0.5 V for a purely resistive electrode with the load impedance determined purely by the spreading resistance for a tissue conductivity of 0.5 S/m.

The current is being delivered into the tissue by disc electrodes, and therefore we will approximate tissue resistance with the well-known expression for spreading resistance:

$$\begin{array}{l} R_{e}=\frac{1}{\sigma d} \end{array}$$

where *d* is the diameter of a disc-shaped electrode, σ is the conductivity of the tissue, and *R*_*e*_ is the spreading resistance of a single electrode. Spreading resistance defines the resistance encountered as the current flows from the very conductive metal electrode (e.g., $\sigma_{platinum}\approx 10^{7\;}$S/m) into the moderately conductive tissue. Estimates of tissue conductivity for gray matter vary, but are generally in the range of 0.1–0.3 S/m at low frequencies (<1 kHz) (Gabriel et al., 2009). However, it is not clear that these measurements of bulk tissue conductivity are representative of the micro-environment around a wireless floating stimulator. For example, extracellular fluid has a much higher conductivity (>1 S/m) than bulk tissue. The spreading resistance of a single electrode is plotted over a range of electrode diameters and tissue conductivities (Figure 3A). The total tissue resistance (R), is the series combination of the spreading resistance from both electrodes (R = 2*R*_*e*_).

Additionally, the electrode-electrolyte interface will introduce some impedance, which is often described with a parallel combination of double-layer capacitance and a charge-transfer resistance (i.e., Faradaic resistance) (Wei and Grill, 2009). We will assume the voltage levels are too low for electrolysis and therefore we will neglect the Faradaic resistance. The double layer capacitance is ~50 μF/cm^2^ for platinum (Geddes, 1997, and unpublished observations). For a disc electrode of diameter 0.4 mm, this corresponds to 65 nF. The impedance of this capacitive interface will depend on the stimulus waveform. If we assume a pulse of 1 ms, then there will be energy out around 1 kHz, and therefore the load of 65 nF will impose 2.4 kΩ. Since we are using roughened platinum with significantly higher capacitance per unit area, the total load will likely be <2.4 kΩ. Taken together, we'll approximate the nominal load resistance to be 10 kΩ, roughly corresponding to two series electrodes of 0.3–0.5 mm with tissue conductivity of 0.5–1 S/m. We will assume the double layer capacitance of the electrodes is negligible when compared to tissue resistance, but it should be noted that this assumption depends heavily on electrode material and on stimulus waveform.

### Defining requirements for current

The neuronal response to electrical stimulation has been extensively studied (Tehovnkik et al., 2006; Freeman et al., 2011). Typically, electrical stimulation is performed using trains of short-duration pulses on the order of 0.1–1.0 ms, with each pulse consisting of a cathodic phase followed immediately by an anodic phase (Cogan, 2008). Importantly, our device delivers only monophasic pulses because of the nature of the rectification circuitry. For example, in order to make our device output a pulse of 0.2 ms, the transmitter will emit a short pulse of AC magnetic field for 0.2 ms, and this signal would be received by and rectified by the stimulator, producing a DC output current lasting 0.2 ms. Because of this design, cathodic pulses will always be delivered from one electrode and anodic pulses from the other electrode. Since neurons are more sensitive to cathodic stimulation, we will consider the cathodic electrode to be the primary means of stimulation.

The threshold for excitation of a single neuron is determined by electrode-to-neuron distance and by current density, defined as current per unit area of the electrode surface (Tehovnkik et al., 2006). Unlike neural implants that are driven by a current source, the input to our stimulator is an EMF induced by a time-varying magnetic field, and therefore the amount of current will depend on the magnitude of the load. The impedance of the load will decrease as the electrodes become larger, which means the total current will increase for larger electrodes. However, the current density will *decrease* as electrodes become larger (Figure 3B). Therefore, our choice of electrode size is a trade-off between achieving the highest current density possible, but also aiming for a maximal area of stimulation to excite as many neurons as possible. We chose electrode sizes on the order of 0.3–0.4 mm in diameter as a tradeoff between: (1) smaller electrodes achieve larger current density levels, and (2) larger electrodes will excite a broader area and therefore will recruit more neurons. Because current spreads out as it leaves the electrode, the electrode-to-nerve distance is critical. In our experiments, we do not have precise control over the distance between the electrode and the nerve, but we expect this distance to be on the order of the electrode diameter (<0.3–0.4 mm), and therefore we expect current spreading to have minimal impact on the observed thresholds. Furthermore, this electrode size has been used in similar work to achieve excitation of peripheral nerves in rodent (Romero-Ortega et al., 2015).

### Surgical procedure and motor evoked response measurements

Four adult Sprague-Dawley rats were used in this study to confirm the ability of the wireless stimulator to elicit action potentials in peripheral nerve axons. The animals were anesthetized with 1.5% isoflurane and the left thigh was shaved and sterilized with 70% ethanol and povidone-iodine. A lateral incision was made in the left hind limb, starting ~2 cm caudal to the hip bone and in a plane parallel to the femur. The vastus lateralis and biceps femoris muscles were separated exposing the sciatic nerve. The wireless stimulator was placed with the cathode facing the nerve and the anode facing the vastus lateralis muscle. All surgical and experimental procedures were approved by, and conducted in accordance with, the ethical guidelines of the UTA and UTD Institutional Animal Care and Use Committee (IACUC).

The motor response was evaluated by placing the antenna 7.5 cm from the nerve. Video recordings were acquired using Plexon CinePlex Studio V3 and OmniPlex acquisition system at 30 frames/sec. Nerve stimulation was tested using square 250 ms pulses at 2 Hz and at various current levels. The evoked limb movement was tracked using ImageJ to obtain the XY position of the paw in several frames. The Euclidian distance with respect to a baseline was calculated for every frame and plotted with Matlab (Mathworks, Natick, MA).

## Results

The wireless neural stimulator (Figure 1) consists of a coil (L) to receive inductive power, a tuning capacitor (C_res_), a diode (D) for rectification, and an optional shunt capacitor (C_shunt_) to facilitate rectification. This circuit is attached to two disc electrodes to provide stimulation to surrounding tissue. We performed computational analysis of wireless energy transfer through inductive coupling in order to define the number of turns required in the coil.

### Computational model of the receive coil

**Figure 4:**
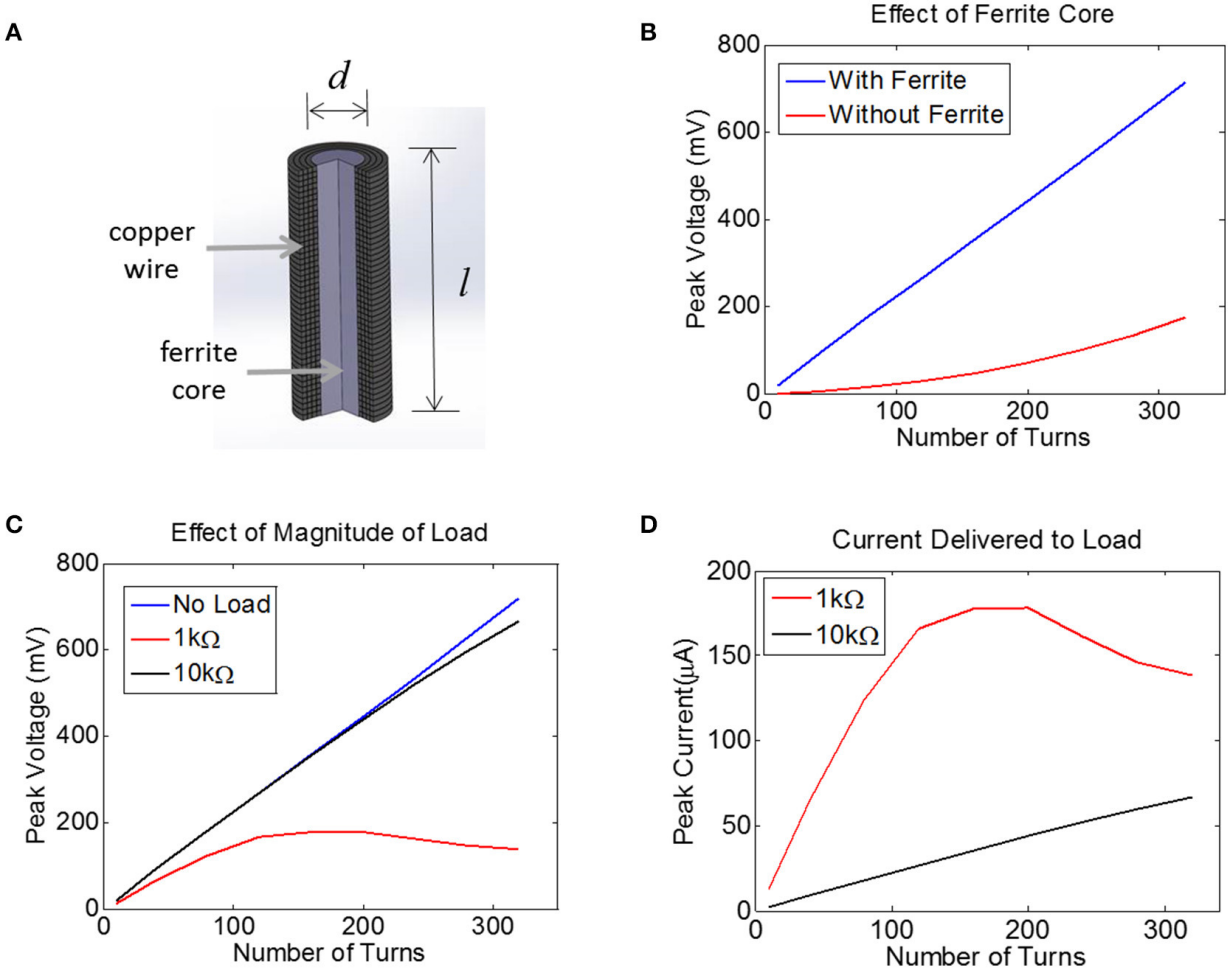
**(A)** Finite-Element Model of a multi-turn coil with ferrite core used to estimate induced voltage in COMSOL. A cross-section is shown to illustrate winding. **(B)** The peak voltage induced in a coil with *d* = 0.2 mm and *l* = 1.0 mm is plotted as a function of the number of turns, both with a ferrite core (blue) and an air core (red), assuming a 10 MHz transmit frequency with the maximum allowed magnetic field of 40A/m, with 52 AWG copper. **(C)** Assuming a 0.2 mm coil with a ferrite core, the peak voltage is plotted for three different load conditions: no load (open circuit, blue), 1 kΩ (red), and 10 kΩ (black). **(D)** For the data in panel **(C)** the peak current is plotted for two different loads, 1 kΩ and 10 kΩ.

For inductive power transfer, there are analytical expressions that can be used to relate the applied AC magnetic field to the induced voltage in the coil. For coils with a high-permeability core, the induced voltage is typically expressed as a linear function of the relative permeability of the core. For example, a relative permeability of 100 should result in an induced voltage that is increased by a factor of 100. In reality, however, the induced voltage does not scale linearly with permeability, but exhibits complex dependencies on geometry (e.g., length-to-width ratio of the core). To account for these dependencies, we built a computational model of a multi-turn coil with ferrite core using COMSOL (Figure 4A). We applied a magnetic field of 40 A/m at 10 MHz to the multi-turn coil and measured the induced voltage (Figure 4B). We used a transmit frequency of 10 MHz because the ferrite core becomes lossy above this frequency. We used a field level of 40 A/m because this is the maximum allowed field level that would be seen by a device that is located on the surface of the biological tissue (2.5 cm from the transmitter), at ~28% input power (Figure 2C). This assumes a SAR limit for an uncontrolled environments (1.6 W/kg, see blue dashed line in Figure 2D).

The voltage induced in the coil was estimated with and without a 0.2 mm-diameter ferrite core. There was no load present (open circuit) for this simulation and thus no current flowed. This means the coil inductance did not play a role. It is clear that the ferrite will significantly boost the voltage on the coil, although the extent of this increase in voltage depends non-linearly on the number of turns.

Next, we attached a resistive load across the terminals of the coil using the COMSOL-SPICE interface in which the two ends of the coil act as terminals of a load that is input to a SPICE model. In our case, these terminals were attached to a resistive load. Results indicate that the induced voltage is little affected for a load of 10 kΩ (Figure 4C). However, for a load resistance of 1 kΩ, the voltage plateaus as the number of turns is increased, as the impedance of the inductor becomes comparable to the load resistance. Despite the fact that the voltage becomes compressed for the 1 kΩ load, the total current is still higher for the 1 kΩ load as compared to the 10 kΩ load (Figure 4D). Note that a 1 kΩ load would allow us to reach the required current of 25 μA with very few turns, but unfortunately the tissue load will likely be closer to 10 kΩ than to 1 kΩ (see Methods). A 10 kΩ load would require between 100 and 150 turns to achieve a peak voltage across the load of 250 mV, corresponding to a peak current of 25 μA. This model illustrates how much voltage will be induced for a coil attached to a resistive load. However, we must also account for the impact that the diode and the tuning capacitor will have on the induced voltage.

### Incorporating a diode and shunt capacitor

The signals that are used to transmit wireless energy are in the radio frequency regime. These frequencies are too high to excite neurons because the voltage-gated ion channels that underlie action potentials operate on the 0.1–1.0 ms timescale (1–10 kHz). Therefore, we need some means of converting energy from high-frequency to low-frequency. The simplest technique to do this is to half-wave rectify the signals with a diode, which will produce output current at DC. However, diodes are not perfect rectifiers since there will be some voltage dropped across the diode itself. To maximize the voltage across the load, we require a diode with the lowest possible turn-on voltage.

**Figure 5:**
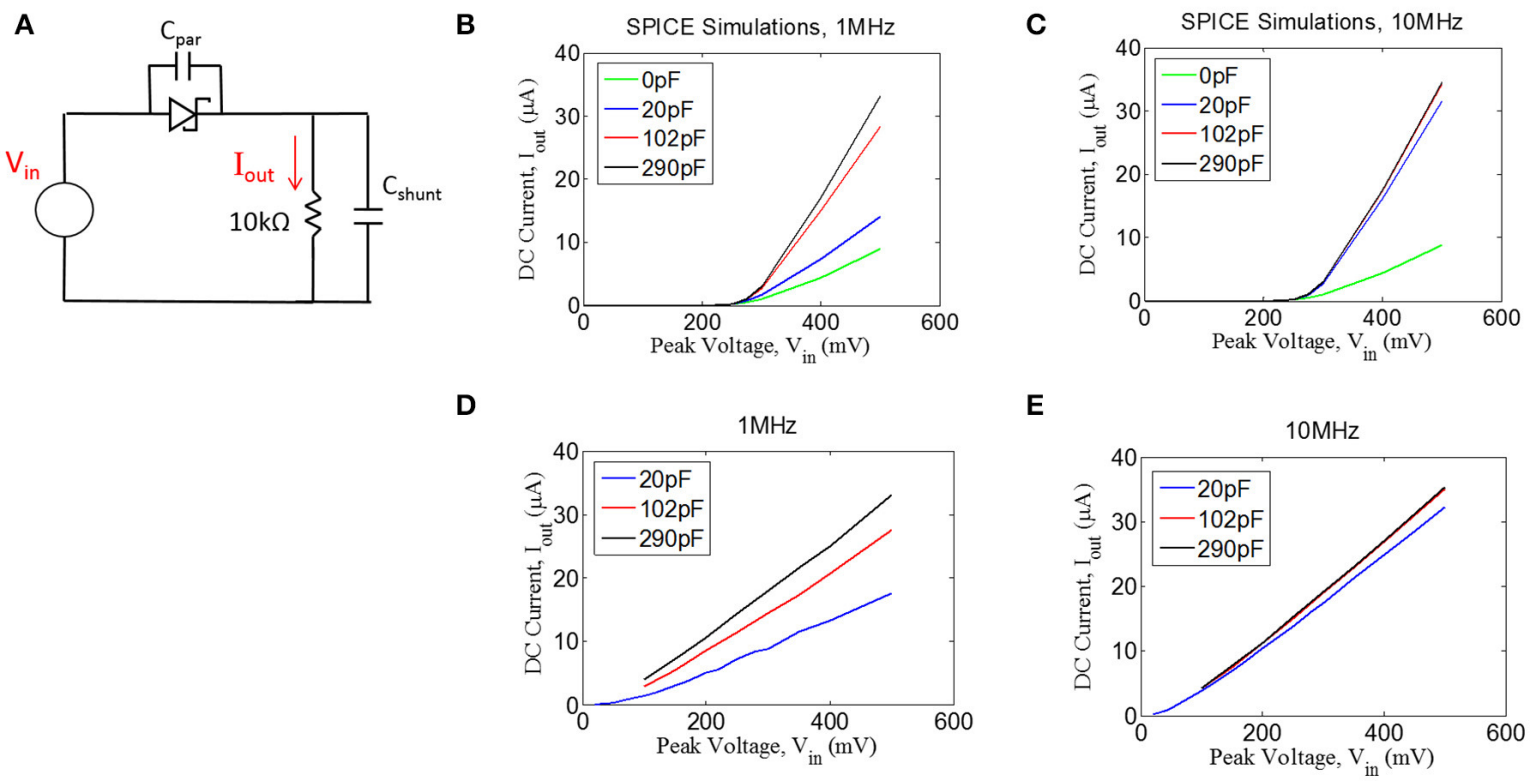
**(A)** Quantifying rectification by applying an AC signal (V_in_) and measuring the time-averaged mean of the current, I_out_, through a resistive load for different values of shunt capacitance (C_shunt_) for 1 MHz **(B,D)** and 10 MHz **(C,E)**. **(B,C)** Spice simulations showing for the circuit shown in **(A)**, assuming a parasitic capacitance of the diode (C_par_) of 0.2 pF. **(D,E)** Experimental measurements with an RF Schottky diode.

We tested the ability of a Schottky diode to rectify by first simulating the circuit shown in Figure 5A (LTSpice, Linear Technology, Milpitas, CA). A voltage source was used to drive the diode into a 10 kΩ load. An ideal diode was used for the simulations with an additional capacitor (C_par_) of 0.2pF placed in parallel to the diode to represent parasitic capacitance (see below). The current through the 10 kΩ resistor was measured, containing both AC and DC components. We have plotted the DC component of the current (I_out_) as a function of sinusoidal input voltage of 1 and 10 MHz (Figures 5B,C). Interestingly, we found that more DC current could be achieved when a shunt capacitor (C_shunt_) was placed in parallel with the resistive load. It is important to note that the role of the capacitor C_shunt_ is not that of a typical smoothing capacitor on a voltage regulator. Rather, this capacitor acts to facilitate rectification by compensating for the parasitic capacitance in the diode; the shunt capacitor was found to have no effect if the diode has zero parasitic capacitance (not shown).

In addition to the simulations, we performed benchtop testing with the test setup shown in Figure 5A. We surveyed various types of diodes for this application. Since traditional Schottky diodes do not conduct until around 300 mV, we chose to use a zero-bias Schottky diode that is designed to conduct near zero voltage. The particular diode chosen is designed for RF applications, having a parasitic capacitance of ~0.2 pF (see Methods). These zero-bias diodes have the drawback of having significant reverse leakage current that is not present in standard Schottky diodes. Despite this drawback, we found that the zero-bias diodes were able to rectify the input signal, as shown in the results in Figures 5D,E. These data matched well with the simulation results, demonstrating that the inclusion of a shunt capacitor will provide a significant improvement in rectification, at least for 1 MHz.

For the 10 MHz case (Figure 5E), it appears as though the impact of the shunt capacitance is minor, but this is primarily because we could not take a measurement for 0 pF of shunt capacitance due to our inability to exclude any parasitic capacitance from the test setup. However, the simulation results at 10 MHz (Figure 5C) suggest that some small amount of shunt capacitance (~10–20 pF) will be necessary to achieve optimal rectification. Future testing will be needed to evaluate whether the tissue itself could produce sufficient capacitance to facilitate rectification, in which case the shunt capacitor could potentially be removed from the design.

### Benchtop testing of fully encapsulated devices

We built a fully encapsulated, functioning device and measured its performance with a series of benchtop tests. The device consisted of a 150-turn coil with 52 AWG wire wrapped around a core of Nickel-Zinc ferrite (#61, Fair-Rite). The coil had an inductance of 31.0 μH, and was tuned to resonate at 10.9 MHz by adding a tuning capacitor of 7.0 pF. The reason for choosing this resonant frequency was that the ferrite becomes significantly lossy for higher frequencies. The device also include an RF Schottky diode and a shunt capacitor of 100 pF. A 10 kΩ resistor was soldered to the platinum disc electrodes during testing to represent the tissue load. The devices were encapsulated in Epoxy (Epo-Tek 301), leaving the disc electrodes exposed. This epoxy was chosen because it has been approved for use by the FDA in medical implants.

**Figure 6:**
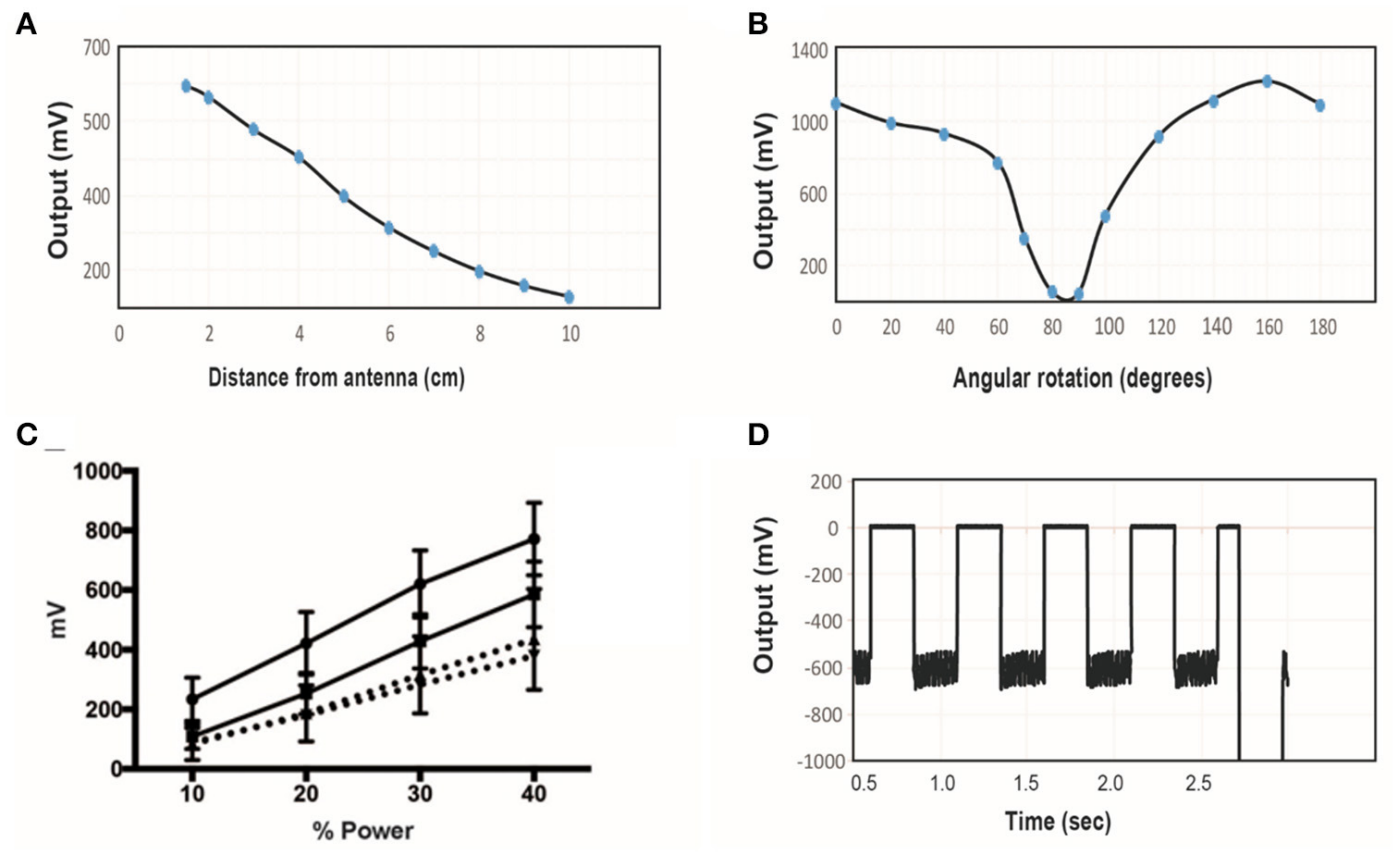
**(A)** The induced voltage in the electroparticle as a function of distance from the transmitter for an input power of 40%. **(B)** Induced voltage at 0 cm distance measured as a function of rotation angle relative to the plane of the transmitter. **(C)** Electroparticle output as a function of applied magnetic field at a distance of 5 cm for four different devices. **(D)** Induced voltage during square-wave excitation during the waveforms used for motor excitation: a train of 1 ms RF-pulses was delivered at 50 Hz for 250 ms, and this was repeated at 2 Hz. The transmit frequency was 10.9 MHz and the load connected across the two disc electrodes was a 10 kΩ resistor in all cases.

A transmitter with diameter of 6″ (15.2 cm) was tuned to 10.9 MHz and the power level was set to 40%. The wireless stimulator was positioned so that the plane of the coil was aligned parallel to the plane of the transmitter. The induced voltage was measured as a function of distance, moving the stimulator along the central axis of the transmitter (Figure 6A). As expected, the induced voltage reaches about one-third of its maximum value at a distance equal to half the transmit diameter (compare Figure 6A to Figure 6B).

The effect of angular rotation of the stimulator with respect to the transmitter showed minor changes in the induced potential up to 60° angle (Figure 6B). When the stimulator was positioned 5 cm away from the transmitter and the intensity of the magnetic field was varied, the induced voltage increase monotonically, as expected (Figure 6C). The stimulus waveform that is used in the animal studies is also shown (Figure 6D), consisting of a train of 1 ms-pulses delivered at 50 Hz. This stimulus was cycled on and off at 2 Hz. These tests show that that the wireless stimulators can produce tens of microamps of current through a 10 kΩ load. Previous studies show that this level of current is sufficient to activate peripheral nerves (Romero-Ortega et al., 2015).

Finally, we performed accelerated lifetime tests to determine whether ingress would occur when the stimulator was exposed to warm saline. Before the tests, the stimulators were confirmed to be functional by performing a diode check between the two disc electrodes with a hand-held multi-meter. Additionally, the device was inspected visually to ensure there were no clear voids within the device. Three stimulators were then placed in 80°C saline for a duration of 2 weeks, and were removed approximately once every 3 days for a diode test. We found that all three devices successfully passed the diode test after 2 weeks, and there was no visible indications of ingress on the devices.

### Evoked nerve stimulation in the acute sciatic nerve rat model

**Figure 7:**
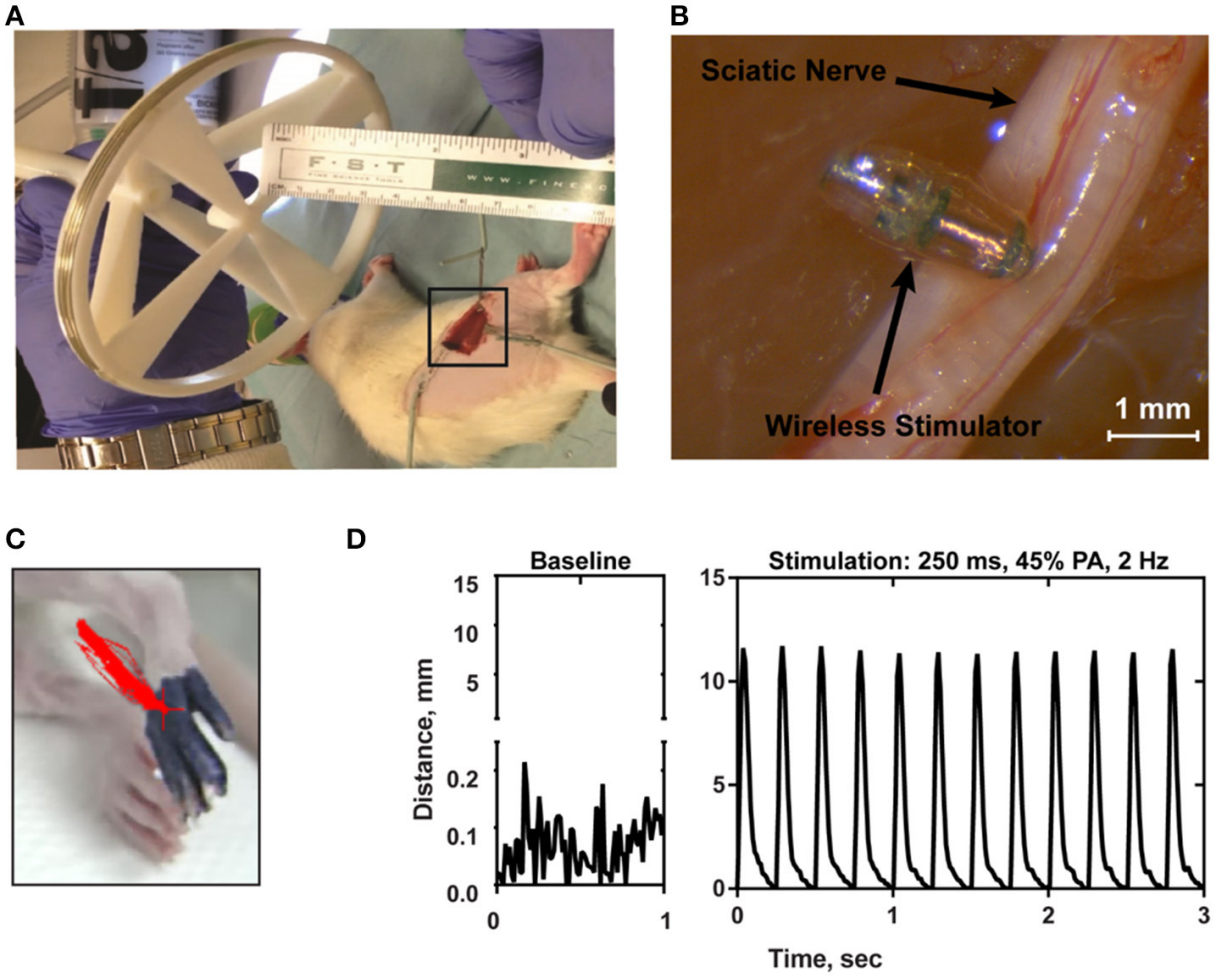
**(A)** Photograph of the transmitter positioned 7.5 cm from the electroparticle on the hindlimb. **(B)** The electroparticle cathode was placed onto the sciatic nerve, and the power amplifier was set to 45% transmit power. Scale bar = 1.5 mm. **(C)** Videoframe of the rat paw with overlaying traces of the evoked movements (red lines) by stimulation. **(D)** Displacement of the hindlimb by a 2 Hz stimulation shows >10 mm evoked movement.

In order to confirm the ability of the wireless stimulator to elicit action potentials in axons, we acutely implanted the stimulator onto the rat sciatic nerve by placing the cathode electrode on the epinerium and the anode to the adjacent muscle (Figures 7A,B). The transmitter was positioned 7.5 cm from the stimulator (Figure 7A). The power level was set to 45%, corresponding to ~20–35 A/m of magnetic field at the location of the stimulator (see Figure 2C). We do not have control over the precise distance between the electrode and the nerve, but given that the thickness of the epineurium in rat sciatic nerve, we estimate this distance to be on the order of hundreds of microns (Navarro et al., 2005). We evaluated limb movement using a high-speed video camera to observe evoked dorsiflexion of the paw. We found that this power level, stimulation of the sciatic nerve was able to evoke a clearly visible movement of the hindlimb in response to a train of 1 ms-pulses at 50 Hz for 250 ms. Figure 7C shows a frame of the paw with a tracing of the evoked movement in one axis. Figure 7D illustrates the baseline movement of the limb prior to stimulation (left), as well as the evoked response with the stimulator on (right), which caused >10 mm displacement. If the orientation of the device was flipped so that the anodic electrode was touching the nerve, then no hindlimb movement was observed (not shown).

**Figure 8:**
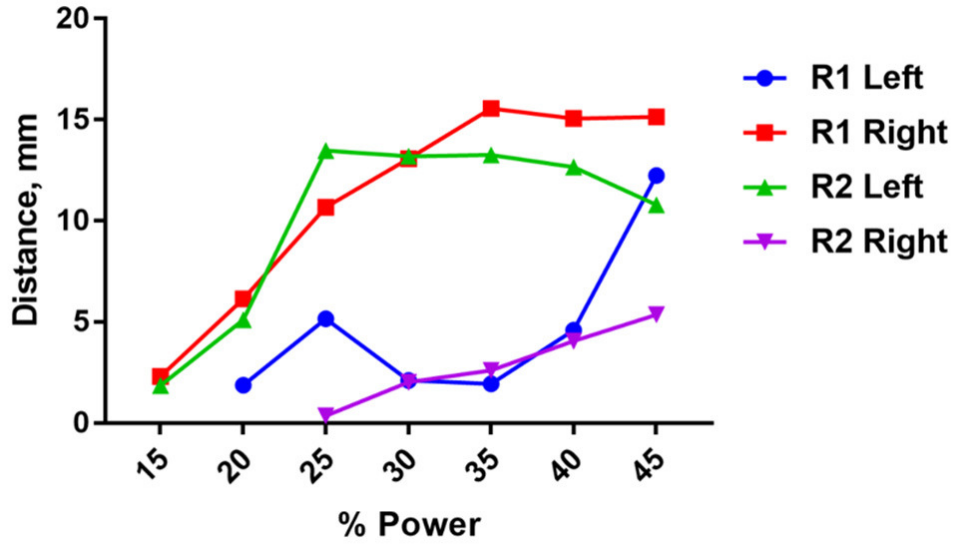
Distance moved by the hindlimb in response to stimulation of the right or left sciatic nerve in two rats (R1 and R2) as a function of input power to the transmit coil. A 10.9 MHz sinusoidal signal was applied to the transmit coil for 1 ms bursts, and these bursts were delivered at 50 Hz for 250 ms. The transmit coil was positioned 7.5 cm from the sciatic nerve.

There was significant variability in the motor responses across trials that was likely due to inconsistent positioning of the stimulator relative to the nerve. This variability was quantified using a single wireless stimulator to stimulate four different nerves, including the left and right nerve of two rodents (Figure 8). While two nerves showed very pronounced movements of >10 mm, the other two nerves showed weaker movements around 2–5 mm (Figure 8). Despite this variability, it was clear in all cases that increasing the stimulation level caused a greater amount of movement. This variability can be mitigated in future iterations of the design by incorporating the device into a nerve cuff that wraps around the nerve, which will be necessary to hold the device in place for chronic implantation of the device. But for the purposes of this study, these results show clearly that robust neural excitation can be achieved with a sub-millimeter inductively powered stimulator.

### Saline testing of monophasic pulsing strategies

Unlike conventional neural stimulators, the output current of the stimulator presented here is always monophasic and cannot be charge balanced by adding a second phase of opposite polarity. In this circumstance, one electrode in the bipolar pair is polarized only in the negative direction and the other only in the positive direction. After the RF power is turned off at the end of a pulse, each electrode remains polarized and discharges over a time course determined by the tissue resistance and electrode capacitance. If the discharge is not complete before the next pulse, the polarization on the electrodes will build to a steady state value determined by the pulsing parameters. The steady state polarization is determined by the pulse parameters and opposing chemical reactions at the electrodes that act to reestablish the equilibrium potential.

We conducted preliminary tests in order to quantify the steady state polarization and the ability of the devices to sustain charge injection. The experiment consisted of 400 μm diameter electrodes subjected to isolated monophasic voltage pulses, similar to those that would be generated by RF excitation of the device. Constant voltage rectangular pulses were applied at a pulse rate of 20 Hz and with pulse widths of 200 and 400 μs using a Tektronix AFB2021 arbitrary function generator to switch a custom optical isolator (Sigenics, Chicago IL). The optical isolator switches a DC voltage source on and off according to the waveform provided by the function generator. Current in response to the applied voltage was measured with a low-noise current preamplifier (Stanford Research Systems SR570). During the measurements, the electrodes were in an inorganic model of interstitial fluid (model-ISF) and the polarization of the electrodes measured against a Ag|AgCl reference electrode.

**Figure 9:**
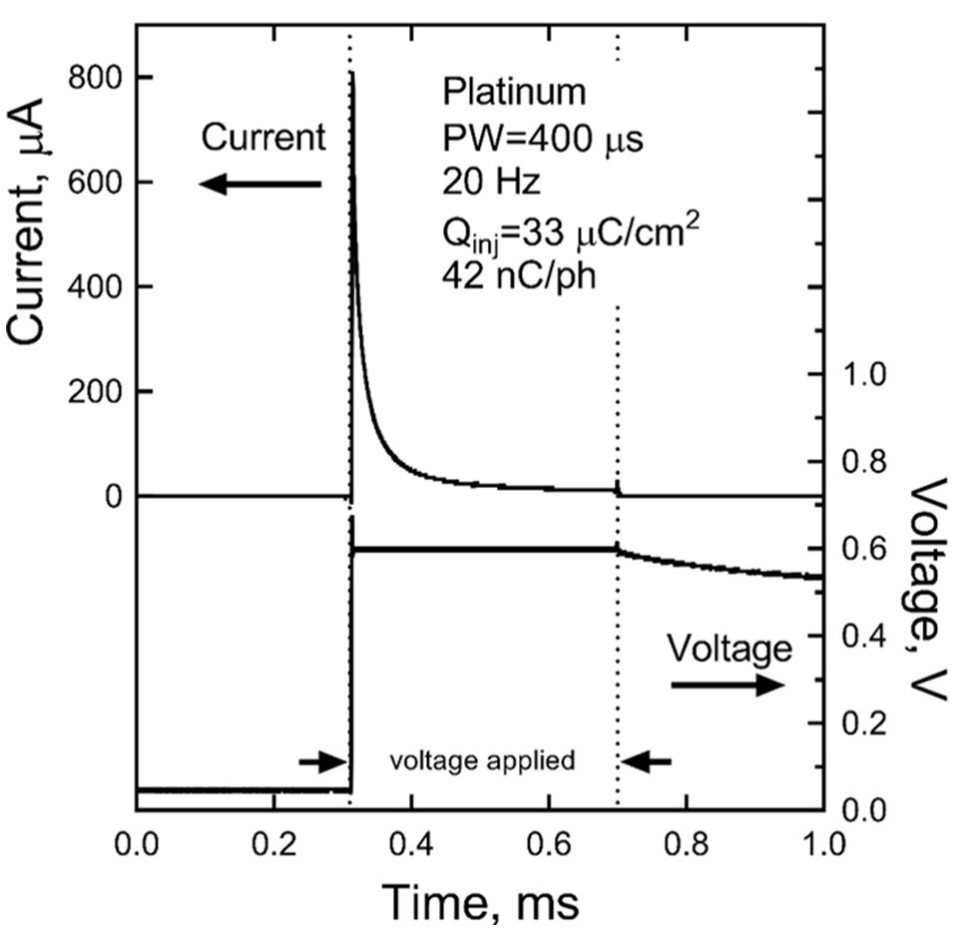
Steady-state current after 300 s of pulsing at 0.6 V and 20 Hz for a platinum bipolar electrode pair. The voltage is applied between 0.3 and 0.7 ms.

The steady-state current, measured after 300 s of pulsing, sustained at a bipolar pair of platinum electrodes in response to a 400 μs, 0.6 V pulse applied at a pulse frequency of 20 Hz is shown in Figure 9. In this data, the voltage is applied between the bipolar pair from 0.3 to 0.7 ms. The slow discharge in the cell voltage can be observed after the applied voltage is turned off. By the next pulse, ~50 ms, the cell voltage has discharged to about 50 mV. Integration of the current response yielded a steady state charge per phase and charge density of 42 nC/ph and 33 μC/cm^2^, respectively. Similar results were obtained for sputtered iridium oxide (SIROF) and porous TiN electrodes, although the current on these electrodes was more constant over the course of the pulse (not shown). The SIROF had modestly higher charge injection capacity (42 nC/ph, 38 μC/cm^2^) whereas the TiN had a lower capacity (33 nC/ph, 26 μC/cm^2^) than the platinum electrode at steady state.

## Discussion

### Reducing antenna size by reducing voltage requirements

We present here a design for a sub-millimeter, inductively powered, wireless neural stimulator. By removing any transistors, we were able to reduce the voltage levels that must be induced in the implanted coil, and with lower voltage requirements, the size of the coil could be reduced. Another way to describe this rationale is that voltage is, by definition, a form of energy (1 Volt = 1 Joule / Coulomb), and if we operate CMOS off of a supply of 1 V, then this voltage represents a potential energy that is put into the FET and is stored in the p-n junction. Importantly, if we are operating off of wireless power, then this power source must provide a drain-source voltage of 1 V in order to bring the FET out of the triode region and into saturation, and this is true even when zero power is actually being dissipated as heat (e.g., for zero gate-source voltage, no power is actually dissipated, even though energy is stored in the p-n junctions). Our device simply removes the requirement to provide this level of drain-source voltage because we don't have any FETs. Because we have relaxed the voltage requirements, we can now make a smaller coil, and this is what enabled the small size of our device.

### Clinical and research applications for wireless neural stimulators

There are a number of clinical and research applications where this stimulator could be useful. Given the growing interest in the commercialization of peripheral nerve therapies (Famm, 2013), we envision this device being integrated into a nerve cuff. This cuff will wrap around the target nerve and provide electrical stimulation when powered by the external transmitter. For example, treatments for urinary incontinence or chronic migraines could be delivered intermittently by the patient, at home, with a hand-held transmitter. Future testing will be needed to evaluate the extent to which these conditions will respond to intermittent stimulation as opposed to current approach, which often involves continuous stimulation (Noblett and Cadish, 2014). Another factor that must be considered for translation to human peripheral nerves is that when this device is integrated into a nerve cuff, there is the potential that the electrode-to-nerve distance will be larger than in our experiments in which we directly placed the electrode onto the epineurium. This increased distance would increase the threshold necessary for neural excitation. To mitigate this, future designs could involve larger coils in order to provide more current; this will come at the expense of increased device volume.

Therapies involving deep brain stimulation (DBS) could benefit from wireless neural stimulators. For example, totally wireless implants could reduce the level of scar tissue growth that results from tethered leads. However, clinical applications of DBS would be challenging because of the difficulty associated with removing the device in the case of infection. We did not investigate removal strategies in this study. More likely, the device presented here could find applications in animal studies on DBS where tethering the animal is undesirable. This is particularly true for behavioral assays that model aspects of human mental illness using DBS-like stimulation during monitoring of animal behavior (Hamani and Temel, 2012). These experiments often require head-fixation and/or the use of bulky tethers, either of which can limit the full expression of natural behavior, may cue the animal to adopt new behavior patterns, and often limit stimulation to at most a few hours in a special test cage. Human DBS, by contrast, is delivered continuously in a natural environment replete with social interactions and complex decisions. Continuous yet tether-free stimulation could substantially improve the translational relevance of animal models.

### Alternative approaches to wireless neural stimulation

There have been many designs of wireless neural stimulators that are relatively large in size (>1 mm^3^) (Okabe et al., 2015; Zargham and Gulak, 2015; Larson and Nurmikko, 2016). The well-known RF BION uses inductive coupling at 2 MHz with a multi-turn coil with ferrite, but is ~10 times larger than our device, measuring 16 mm in length and 2 mm in diameter (Loeb et al., 2001). Another inductively coupled stimulator that was also designed using a simple rectifier included planar coils of 60 mm in diameter, which is considerably larger than the device presented here (Ha et al., 2012). Other stimulators have used higher frequencies for energy transmission, such as a microwave powered stimulator (915 MHz) that measures 10 mm in length and 0.8 mm in diameter (Towe et al., 2012). Another recent design uses so-called mid-field coupling at 1.6 GHz, resulting in a device that is about 5 mm in the longest dimension (Ho et al., 2014). Optically powered stimulators have been developed using photodiodes that are extremely small in scale (<0.01 mm^3^), but this approach works only at superficial depths due to the inability of light to penetrate tissue (Abdo et al., 2011; Seymour et al., 2014). This issue with light penetration also presents a challenge for optogenetically modified neurons that can be made sensitive to light (Boyden, 2011).

Nanoparticle-mediated stimulation is an attractive approach if nanoparticles can be delivered through the blood, avoiding the costs and risks of neurosurgery. But even if nanoparticles are designed to be able to cross the blood brain barrier, two challenges remain. First, the inability to control the location of the nanoparticles within the brain will result only in widespread activation (Yue et al., 2012), and therefore may not offer improvements over transcranial magnetic stimulation in terms of the spatial pattern of excitation. Secondly, the amount of nanoparticles needed for excitation can be quite large (Chen et al., 2015), requiring that the nanoparticles are injected directly into the brain rather than through intravenous injection. Another set of studies has attempted to wireless magnetic stimulation with ferritin as a transducer (Stanley et al., 2012; Wheeler et al., 2016), but the interpretation of the results are still under debate (Meister, 2016).

### Monophasic stimulation

Monophasic stimulation without active charge-balance is atypical in neural stimulation. The result is residual polarization of the electrode that slowly discharges during the period between pulses. The limits to monophasic pulsing in terms of deliverable charge and electrode stability are currently being investigated. The preliminary results reported here show that it is possible at steady state (300 s) to sustain modest levels of charge injection (30–40 nC/ph) with 400 μm diameter electrodes. Charge densities increase with decreasing pulse frequency and are higher for shorter periods of pulsing. More detailed characterization of Pt, SIROF, and TiN electrodes under a broad range of monophasic pulsing conditions obtainable with the present wireless stimulation device is ongoing. A significant risk of monopolar stimulation is tissue damage induced by electrode reactions. Measurements of platinum electrode potentials (vs. Ag|AgCl) indicate that the electrodes remain well-within water electrolysis limits during monophasic pulsing with the parameters reported in Figure 7. However, histological measures of tissue damage in response to chronic stimulation will be required to assess the safety of wireless monophasic approach.

### Risks associated with the wireless stimulator

One of the risks that was not evaluated in this study that tissue encapsulation will increase the tissue impedance enough that the device can no longer deliver therapeutic levels of stimulation. The extent to which tissue encapsulation influence electrode impedance is difficult to predict because there is significant variability in the literature (Ward et al., 2009). Chronic studies with microelectrodes used for recording only, without stimulation, show rapid increases of impedance developing over several weeks after implantation (Prasad and Sanchez, 2012). Other chronic studied have used microelectrodes to stimulate as well as record have found that after an initial period increased electrode impedance, there is a gradual return of impedance to initial values after 12 weeks (Davis et al., 2012).

We can roughly estimate the change in impedance due to scar tissue by taking into account two factors: (1) the expected thickness of the encapsulation layer, which may be on the order of 25 μm (Ersen et al., 2015), and (2) the conductivity of the encapsulation layer, estimated to be ~0.15–0.3 S/m (Grill and Mortimer, 1994). Taken together, we can estimate the resistance of a 25 μm thick layer of scar tissue on a 300 μm disc electrode with 0.2 S/m conductivity to be 1.8 kΩ. Conversely, with no scar tissue, assuming a conductivity of 0.5 S/m, the same layer of tissue would impose 0.7 kΩ, resulting in an increase of 1.1 kΩ per electrode due to scar tissue. This is a relatively small change in impedance, but these are estimates only that will require animal testing with chronic implants to accurate measure long-term performance.

## Author contributions

DF, JO', PK, AD, BD, RI, and JB designed the device. SC, MR-O, AH-R, and AK carried out the experiments. LS and JW carried out computational modeling. MA, AM, JC, and AC fabricated the device. RG, BI, JC, AM, SF, SL, and AW contributed to data analysis.

### Conflict of interest statement

The authors declare that the research was conducted in the absence of any commercial or financial relationships that could be construed as a potential conflict of interest.
